# Diffusion controls the ventilation of a Pacific Shadow Zone above abyssal overturning

**DOI:** 10.1038/s41467-021-24648-x

**Published:** 2021-07-16

**Authors:** Mark Holzer, Tim DeVries, Casimir de Lavergne

**Affiliations:** 1 School of Mathematics and Statistics, University of New South Wales, Sydney, NSW Australia; 2 Department of Geography, University of California, Santa Barbara, CA USA; 3 Earth Research Institute, University of California, Santa Barbara, CA USA; 4 LOCEAN Laboratory, Sorbonne Université-CNRS-IRD-MNHN, Paris, France

**Keywords:** Carbon cycle, Physical oceanography

## Abstract

Mid-depth North Pacific waters are rich in nutrients and respired carbon accumulated over centuries. The rates and pathways with which these waters exchange with the surface ocean are uncertain, with divergent paradigms of the Pacific overturning: one envisions bottom waters upwelling to 1.5 km depth; the other confines overturning beneath a mid-depth Pacific shadow zone (PSZ) shielded from mean advection. Here global inverse modelling reveals a PSZ where mean ages exceed 1400 years with overturning beneath. The PSZ is supplied primarily by Antarctic and North-Atlantic ventilated waters diffusing from below and from the south. Half of PSZ waters re-surface in the Southern Ocean, a quarter in the subarctic Pacific. The abyssal North Pacific, despite strong overturning, has mean re-surfacing times also exceeding 1400 years because of diffusion into the overlying PSZ. These results imply that diffusive transports – distinct from overturning transports – are a leading control on Pacific nutrient and carbon storage.

## Abstract (web summary)

The deep North Pacific is the end of the road for global ocean circulation, but the circulation patterns and ventilation are poorly understood. Here the authors show that diffusive transports both along and across density layers play a leading role in returning 1,400 year old water to the surface.

## Introduction

The world’s oldest seawater can be found in the mid-depth North Pacific where estimates of the ideal mean age exceed 1300 years^1–4^. The mid-depth North Pacific thus constitutes a vast reservoir of remineralized nutrients and respired carbon accumulated over a long time^5,6^. When the waters of this reservoir are returned to the surface, their high nutrient content supports biological production and their respired carbon can outgas to the atmosphere. While the ventilation of the deep Pacific is thus important for ocean biogeochemistry and climate, there is a great deal of uncertainty about the pathways and rates with which the deep Pacific and the surface ocean exchange water, with divergent schools of thought on the nature of the deep Pacific overturning circulation^7,8^.

The global pathways of newly formed deep water, and the mechanisms through which dense deep waters gain buoyancy to return to the surface, have long been of fundamental interest in oceanography. The observed radiocarbon age of water and simple mixing analyses of hemispherically distinct quasi-conservative properties have led to the concept of the great ocean conveyor^9–11^. This concept is epitomized by a schematic of the large-scale interbasin overturning circulation that has since been elaborated and refined based on absolute geostrophic transports^7,12^ and more comprehensive inverse modelling of observed tracer properties^13–15^. Schematics of the global overturning circulation have become an entrenched conceptual framework for discussing the ocean circulation and its climate-driven changes. However, these schematics are widely acknowledged to be an oversimplification of oceanic transport^7,10,11,16^, in large part due to their inability to adequately capture the role of eddy-diffusive mixing across different branches of the overturning circulation and between vigorously flowing and more stagnant regions such as the deep Pacific. An analysis of the local fraction of water in transit between specified surface regions in ocean models^17–19^ revealed that the pathways depicted in typical overturning schematics only capture the paths that are fast on the scale of the most probable interior residence time and that these advectively dominated fast paths carry typically much less than half the formation-to-reexposure flow rate. The bulk of the flow is carried by paths that explore much larger fractions of the ocean volume, and asymptotically slow paths are organized into the deep North Pacific by what has been dubbed the *diffusive conveyor*^17^.

**Fig. 1:**
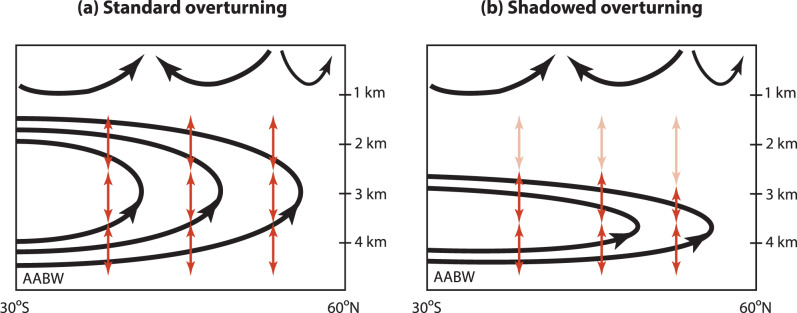
Paradigms of the Pacific meridional overturning circulation (PMOC). Schematic of the PMOC as envisioned by the standard conveyor paradigm (**a**) and by the shadowed conveyor paradigm (**b**). PMOC streamlines are black and diapycnal diffusion is indicated by double-headed red arrows. The deep overturning cell carries Antarctic Bottom Water (AABW) northward. For the shadowed conveyor, the deep overturning is vertically compressed and separated from the mode/intermediate-water circulation of the upper ocean by a shadow zone characterized by relatively weak vertical mixing (light-red arrows).

There are currently two paradigms^20^ for the deep overturning of the Pacific (Fig. 1). The “standard conveyor” paradigm envisions Antarctic Bottom Water (AABW) upwelling and transforming to Pacific Deep Water (PDW) before flowing back south to the Southern Ocean. (The PDW core is considered^7^ to lie at a neutral density^21^ of *γ* = 27.8 kg m^−3^ or roughly 1.6 km depth north of 35^∘^S.) Although this paradigm does not commit to a definite latitudinal distribution of the upwelling AABW, its most recent schematic^7^ and its precursors^10,11^, as well as results from tracer inversions^13–15^, suggest a broad overturning cell that reaches above 2.5 km depth and into the Northern Hemisphere (Fig. 1a). (Geostrophic inverse models at 32^∘^S in the Indian Ocean similarly suggest an overturning circulation with a southward return above ~2000 m depth or higher^22^.) By contrast, it was recently argued^8^ that the area of seafloor intersected by a given density class is a key control on buoyancy-flux divergences and hence overturning. Because 85% of the seafloor lies at depths greater than 2.5 km, this argument implies a vertically compressed abyssal Pacific overturning with southward return flow below ~2.5 km depth (Fig. 1b). This theory hence predicts a weakly ventilated Pacific “shadow zone” that is isolated from the large-scale overturning by being sandwiched north of 32^∘^S between the abyssal overturning cell and the upper wind-driven thermocline circulation. We refer to this view of the Pacific overturning as the “shadowed conveyor” paradigm.

Here we investigate the overturning circulation and ventilation of the deep Pacific using a steady global Ocean Circulation Inverse Model (OCIM^2,23^) that assimilates the observed global distributions of potential temperature, salinity, CFC-11, CFC-12, natural radiocarbon (Δ^14^C) and *δ*^3^He, plus sea-surface height, and surface fresh-water and heat fluxes (see Methods). Due to the inclusion of multiple tracers, as well as dynamical constraints^2^, the OCIM is much better constrained than geostrophic box inverse models^13–15,24^ constructed from hydrographic sections. The OCIM’s high fidelity to the observed tracer distributions and its matrix formulation of the advective-diffusive flux-divergence operator, which allows efficient implementation of Green-function-based diagnostics (see Methods), makes it one of the best available tools for investigating oceanic transport.

For this study, we have doubled the OCIM’s vertical resolution to 48 layers to better resolve deep flows, and we prescribe a spatially varying diapycnal diffusivity *κ*_⊥_ based on tidal mixing^25^ while optimizing the local isopycnal eddy-diffusivity *κ*_∥_ together with the resolved velocity field. *κ*_⊥_ has a large dynamic range with local values below the mixed layer from 1.4 × 10^−7^ to 7.7 × 10^−2^ m^2^ s^−1^, an average above 3000 m depth of 0.31 × 10^−4^ m^2^ s^−1^, and an average below 3000 m of 1.6 × 10^−4^ m^2^ s^−1^. *κ*_∥_ ranges from 134 ± 2 to 4108 ± 28 m^2^ s^−1^ with a global mean of 647 ± 16 m^2^ s^−1^. *κ*_∥_ exceeds 2000 m^2^ s^−1^ in strongly baroclinic regions broadly consistent with observation-based estimates^26–28^ and high-resolution modelling^29^ (Supplementary Note and Supplementary Fig. 1). The tidal mixing parameterization of *κ*_⊥_ includes both bottom-intensified local mixing and stratification-dependent remote mixing^25^; the latter has recently been shown to be important for modelling a realistic ocean radiocarbon distribution^30^ and by implication deep-ocean ventilation timescales. Bottom-intensified mixing is essential for the OCIM to capture a well-defined shadow zone with vertically compressed abyssal overturning beneath, consistent with the theory^8^ that diapycnal mass-flux divergences are driven by exposure to the seafloor.

To assess the sensitivity of our results to the values of *κ*_⊥_, we consider three ocean states: a base state optimized using *κ*_⊥_ as recently mapped and successfully compared to available turbulence observations^25^, and two optimized perturbed states for which this *κ*_⊥_ is either uniformly scaled up or down by a factor of 2. (This four-fold range of *κ*_⊥_ brackets the total power input to small-scale turbulence in the ocean interior^31^ from 0.5 to 2.0 TW, making a significantly larger range for *κ*_⊥_ in the global mean unlikely.) The uncertainty of any quantity *X* reported here is the half-range spread $[\max (X)-\min (X)]/2$ for the $\min$ and $\max$ of *X* across the three states. Note that because the OCIM is optimized for each state to fit the observational constraints as closely as possible, the sensitivities quantified here are smaller than the sensitivities to the same changes in *κ*_⊥_ expected for a forward ocean model. Based on the OCIM circulation, we define shadow and abyssal zones of the North Pacific and systematically quantify the advective-diffusive transport that ventilates these zones and re-exposes their water back to the atmosphere.

We find that both paradigms of the overturning capture aspects of the meridional mean transport of the Pacific, but neither gives a completely accurate picture. In accord with the shadowed conveyor paradigm, the deep overturning is vertically compressed with a well-defined overlying shadow zone, but the shadow zone gradually erodes with decreasing latitude into the Southern Hemisphere. The standard conveyor, if broadly interpreted as representing Lagrangian diffusive paths, captures the diapycnal transport through the Pacific shadow zone, but does not capture the shadow zone itself or the vertically compressed overturning beneath. Here, we provide a more complete picture that quantifies the importance of both Southern Ocean and North Atlantic ventilated waters in supplying the Pacific shadow zone. Our results highlight that the subarctic Pacific, in addition to the Southern Ocean and tropical Pacific, is an important region for re-exposing shadow-zone water back to the atmosphere. A quantification of the surface-to-interior and interior-to-surface path densities, transport rates, and associated mean transit times allows us to contrast a diffusively ventilated shadow zone with the advectively ventilated abyssal region beneath.

## Results

### Overturning, mean ventilation times, shadow and abyssal zones

**Fig. 2:**
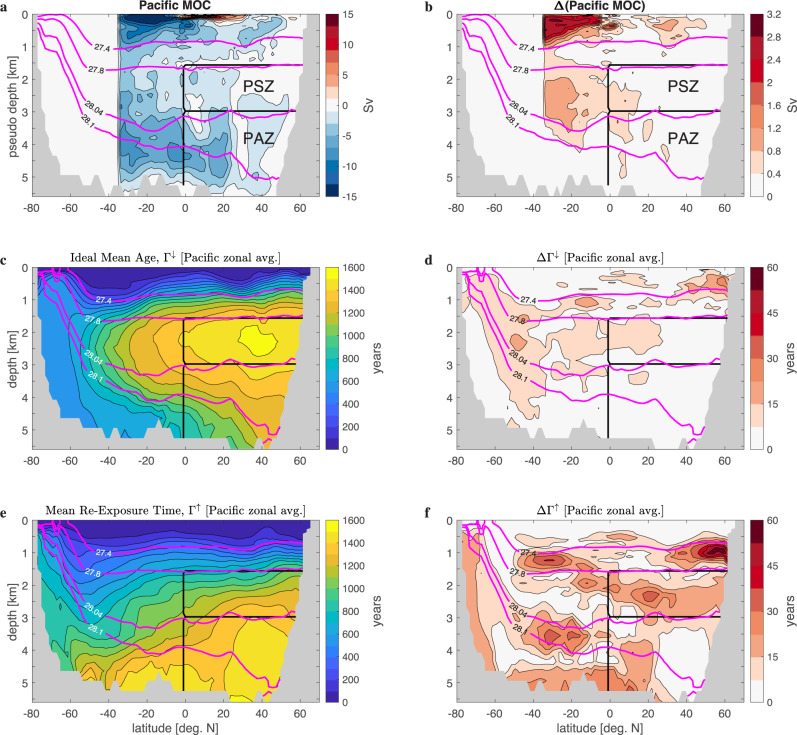
Pacific meridional overturning stream function, ideal mean age, and mean re-exposure time. Stream function of the Pacific meridional overturning circulation (MOC) computed from the meridional flow below a given neutral density and remapped to a pseudo depth^32^ for the Pacific (**a**) and its half-range Δ (**b**) over the three states considered. Pacific zonal averages of the mean time since last surface contact (ideal mean age, Γ^*↓*^) (**c**) and the mean time to next surface contact (mean re-exposure time, Γ^*↑*^) (**e**), and their half-ranges ΔΓ^*↓*^ (**d**) and ΔΓ^*↑*^ (**f**). The heavy black lines delineate the Pacific Shadow Zone (PSZ) and the Pacific Abyssal Zone (PAZ) beneath. Because the North Pacific between 1500 and 3000 m depth is characterized by both weak overturning and the oldest ideal mean ages, we define both the PSZ and PAZ to lie in the North Pacific bounded to the south by the equator. The magenta lines indicate zonal mean neutral density surfaces (contour labels in units of kg m^−3^).

The OCIM-optimized Pacific meridional overturning circulation (PMOC, Fig. 2a, computed as the meridional flow at neutral densities greater than a specified value and mapped back to depth-latitude space^32^) has a deep cell with northward streamflow of AABW well into the Northern Hemisphere; abyssal inflows and outflows across the equator are roughly 8 Sv. The maximum deep overturning of 8.6 ± 0.3 Sv (1 Sv = 10^6^ m^3^ s^−1^) at ~6^∘^S and neutral density *γ* = 28.12 kg m^3^ is consistent with the estimate of 10.6 ± 1.7 Sv for flow through the Samoan and adjacent passages based on moorings and hydrography^33–36^, but significantly smaller than the ~14 Sv of AABW inflow inferred from absolute geostrophic velocities^7,37^. Contrary to the standard conveyor paradigm, the deep overturning cell is vertically compressed and does not feature southward flow centred around *γ* = 27.8 kg m^−3^. In broad agreement with theoretical predictions based on density transformation driven by exposure to the seafloor^8^, the change in direction from abyssal northward flow to overlying southward flow lies around *γ* = 28.1 kg m^−3^, and the southward mean flow north of ~30^∘^S is approximately confined to depths below 2.5 km. Note that both paradigms allow a few Sv of upwelling into the thermocline circulation. Here, the vertical streamflow across 2.5 km depth is generally weak but by ~30^∘^S approaches roughly ~4 Sv, reaching as high as *γ* = 27.4 kg m^−3^ and onward into the thermocline. The sensitivity of the PMOC to a factor-of-2 change in *κ*_⊥_ leads to a PMOC half-range (Fig. 2b) that is less than 1 Sv below 1 km depth, but reaches ~3 Sv in the vigorous southern thermocline circulation.

The North Pacific between roughly 1.5 and 3 km depth is largely shielded from the PMOC and can thus be considered a shadow zone^8^ of the large-scale streamflow, consistent with the shadowed conveyor paradigm. This shadow zone is expected to be weakly ventilated as can be quantified by the ideal mean age, Γ^*↓*^, the mean time since ventilation anywhere at the surface: The Pacific zonal mean of Γ^*↓*^ has its maximum in the shadow zone and its 1400-year contour roughly circumscribes it (Fig. 2c). This region is also where the ocean’s oldest radiocarbon ages Γ_C_ are observed^5,38,39^; Γ_C_ closely approximates Γ^*↓*^ in the deep ocean when corrected for the distribution of Γ_C_ at the surface^40^. Beneath the shadow zone, Γ^*↓*^ decreases by several hundred years with depth and latitude, consistent with better ventilation through coherent AABW access from the south. Changing the magnitude of *κ*_⊥_ by a factor of 2, and re-optimizing, changes Γ^*↓*^ by less than 1% (Fig. 2d), consistent with the high-fidelity fits to observed Δ^14^C (see Methods).

While Γ^*↓*^ provides a measure of the degree of isolation from the overturning, the mean time to next ventilation anywhere at the surface (mean re-exposure time, Γ^*↑*^, Fig. 2e) has a qualitatively different pattern in the deep Pacific highlighting the asymmetric nature of advective-diffusive transport. Water in the abyssal North Pacific is shielded the longest from next ventilation, with a zonal-mean Γ^*↑*^ in excess of 1400 years. This shows that the deep overturning circulation is not efficient in returning AABW back to the surface. As newly ventilated AABW is advected into the abyssal North Pacific, a significant portion (quantified below) diffuses upward out of the direct influence of the overturning, thus imparting these waters with the ocean’s longest mean re-exposure times. The local mean residence time of water in the ocean interior (i.e., the mean time between successive ventilations) is given by Γ^*↓*^ + Γ^*↑*^ (not shown), which has only weak vertical gradients in the North Pacific below roughly 1.5 km depth. The sensitivity of Γ^*↑*^ to changes in *κ*_⊥_ (Fig. 2f) is roughly 4 times larger than for Γ^*↓*^, underlining that tidal mixing is a key control on buoyancy gain and return to the surface.

For a quantitative analysis of the ventilation and re-exposure rates and pathways of the deep North Pacific, we define the Pacific Shadow Zone (PSZ) as the region of the North Pacific bounded horizontally by 1.5 and 3 km depth and vertically by the equator, and we define the Pacific Abyssal Zone (PAZ) as the region immediately beneath the PSZ (Fig. 2). We do not extend the PSZ into the Southern Hemisphere to minimize advective transport into and out of the PSZ and to better align its location with tracer properties such as the ideal mean age Γ^*↓*^.

### Surface-to-interior and interior-to-surface fluxes and mean transit times

**Fig. 3:**
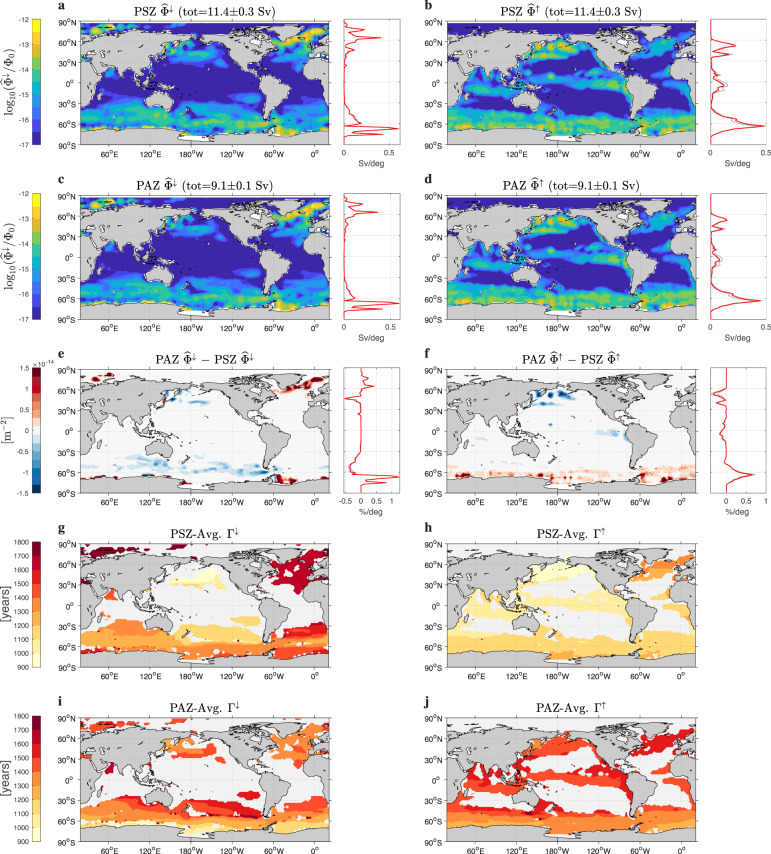
Surface-mapped ventilation and re-exposure rates and mean transit times. The normalized ventilation flux ${\widehat{{{\Phi }}}}^{\downarrow }$ from the surface to the Pacific Shadow Zone (PSZ) (**a**) and from the surface to the Pacific Abyssal Zone (PAZ) (**c**). The normalized re-exposure flux ${\widehat{{{\Phi }}}}^{\uparrow }$ from the PSZ to the surface (**b**) and from the PAZ to the surface (**d**). Because the dynamic range of these fluxes spans several orders of magnitude, plotted are the base-10 logarithm of ${\widehat{{{\Phi }}}}^{\downarrow }/{{{\Phi }}}_{0}$ and ${\widehat{{{\Phi }}}}^{\uparrow }/{{{\Phi }}}_{0}$, where Φ_0_ = 1 m^−2^. The total surface-integrated fluxes in units of Sv = 10^6^ m^3^ s^−1^ are indicated in the plot titles. The difference between the PAZ and PSZ normalized ventilation fluxes (**e**) and between the PAZ and PSZ normalized re-exposure fluxes (**f**). The side panels of (**a**–**h**) show the global zonal integrals (not normalized) per degree of latitude. (Unperturbed base state in red, sensitivity perturbations in grey.) The mean transit times Γ^*↓*^ from a given surface point to an interior point, and the mean transit times Γ^*↑*^ from an interior point to a given surface point, averaged over the PSZ (**g**, **h**) and over the PAZ (**i**, **j**). Γ^*↓*^ and Γ^*↑*^ are plotted only for normalized fluxes (unity surface integral) larger than 0.5 × 10^−16^ m^−2^ because of numerical issues in calculating the mean transit times for smaller flow rates.

Maps of the surface ventilation flux Φ^*↓*^ of water destined for a given interior zone (Fig. 3a, c) and of the surface re-exposure flux Φ^*↑*^ of water from a given interior zone (Fig. 3b, d) reveal that to first order the conduits into and out of the deep North Pacific are very similar for the shadow and abyssal zones. Before examining these fluxes further, it is important to distinguish them from the net volume fluxes across the base of the mixed layer. Here we calculate the flux of water in transit since last contact with a specified origin region (a surface grid box for Φ^*↓*^ and an interior zone for Φ^*↑*^) that is bound to make next contact with a specified destination region. To avoid overcounting, we exclude fluid elements that make repeat contact with the origin region or make contact with the sea surface along the way (see Methods). Importantly, these fluxes are thus one-way (or gross) fluxes^41,42^ that are more useful ventilation metrics than the net volume fluxes because the one-way fluxes govern surface–interior tracer exchange which can be non-zero even for zero net flux^43^.

The pattern of the ventilation flux Φ^*↓*^ is dominated by the high-latitude deep-water formation regions: The subpolar North Atlantic and Nordic Seas, and the Weddell and other Antarctic marginal seas. The pattern of the re-exposure flux Φ^*↑*^ is largest in the Southern Ocean, in regions of tropical upwelling, and in the subarctic Pacific (re-exposure in the subpolar North Atlantic is 3–4 orders smaller; the fluxes in Fig. 3a–d are plotted with a logarithmic color scale). The globally integrated surface-to-interior flow rate, balanced exactly for our steady-state circulation by the corresponding interior-to-surface flow rate, is 11.4 ± 0.3 Sv for the PSZ and 9.1 ± 0.1 Sv for the PAZ.

To reveal the differences between the PAZ and PSZ surface fluxes, Fig. 3e shows the PAZ–PSZ difference of the normalized ventilation fluxes, ${{\Delta }}{\widehat{{{\Phi }}}}^{\downarrow }$, and Fig. 3f shows the differences of the normalized re-exposure fluxes, ${{\Delta }}{\widehat{{{\Phi }}}}^{\uparrow }$. Normalization of the fluxes (by dividing by their global surface integral) is necessary to eliminate the effects of the different interface areas of the interior zones; for example, if all else were equal, we would expect larger total flow rates for the PSZ merely because it can be accessed from below, while the bottom of the PAZ is blocked by the seafloor. The normalized differences show that on a per-Sverdrup basis, the deeper, denser PAZ is preferentially (i.e., relative to the PSZ) ventilated in the deep-water formation regions, while the less dense PSZ is preferentially ventilated where subpolar mode and intermediate waters form. On a per-Sverdrup basis, the PAZ is preferentially re-exposed to the atmosphere in the Southern Ocean south of roughly 45^∘^S, while the PSZ is preferentially re-exposed via upwelling in the tropical Indo-Pacific and in the subarctic Pacific (where there is a shallow overturning circulation). While revealing, the differences between the normalized flux patterns are small, with peak differences in the zonally integrated patterns on the order of 1% of the total global flux per degree latitude. This underlines that the geographic locations of the conduits into and out of the ocean interior are to first order determined by upper-ocean dynamics; deep-ocean destination or origin is of secondary importance.

The mean surface-to-interior transit times Γ^*↓*^ and mean interior-to-surface transit times Γ^*↑*^, averaged over a given interior zone, are mapped out in Fig. 3g–j. The mean transit times Γ^*↓*^ from the North Atlantic surface into the PSZ are about 1700 years, roughly 300 years older than the mean transit time from the North Atlantic into the PAZ, which underscores the fact that (as quantified below) roughly half the water last ventilated in the North Atlantic that enters the PSZ does so by diffusing up into the PSZ while traversing the PAZ. By the same token, the mean transit time from the Antarctic margin, where AABW forms, to the PAZ is around 1100 years, but to the PSZ it is 1400–1500 years. Mode/intermediate waters from the subpolar Southern Ocean reach the mid-depth PSZ on average in 1000–1200 years, which is roughly 200–400 years more quickly than their mean transit into the deeper PAZ (Fig. 3g, i). There is less geographic variation in the mean interior-to-surface transit times Γ^*↑*^. Although only about 1% of the waters of either zone is re-exposed in the North Atlantic, the mean re-exposure times to the North Atlantic, remote from the North Pacific, are the longest for the PSZ (roughly 1300 years, Fig. 3h) and comparable to the transit times of equatorial re-exposure for the PAZ (roughly 1500 years, Fig. 3j). Re-exposure of PSZ waters in the subarctic Pacific, tropical oceans, and Southern Ocean are around 1000–1200 years, while the corresponding re-exposure times for the PAZ are about 400 years longer in the tropical and North Pacific and about 200 years longer in the Southern Ocean, again consistent with re-exposure of dense PAZ waters involving additional slow upward diffusion relative to the PSZ.

### Origin/destination partitioned transport rates and volumes

Despite small streamflow through the PSZ, there can be substantial eddy-diffusive transport into and out of the PSZ if there are gradients in the transported property across its boundaries. The property relevant for our analysis is the concentration of the subset of fluid elements that was labelled in its origin region and whose origin label is removed on contact with the destination region (and on contact with the surface and on repeat contact with the origin region), as we track water from the surface to its first contact with an interior zone or from an interior zone back to the surface (see Methods).

**Fig. 4:**
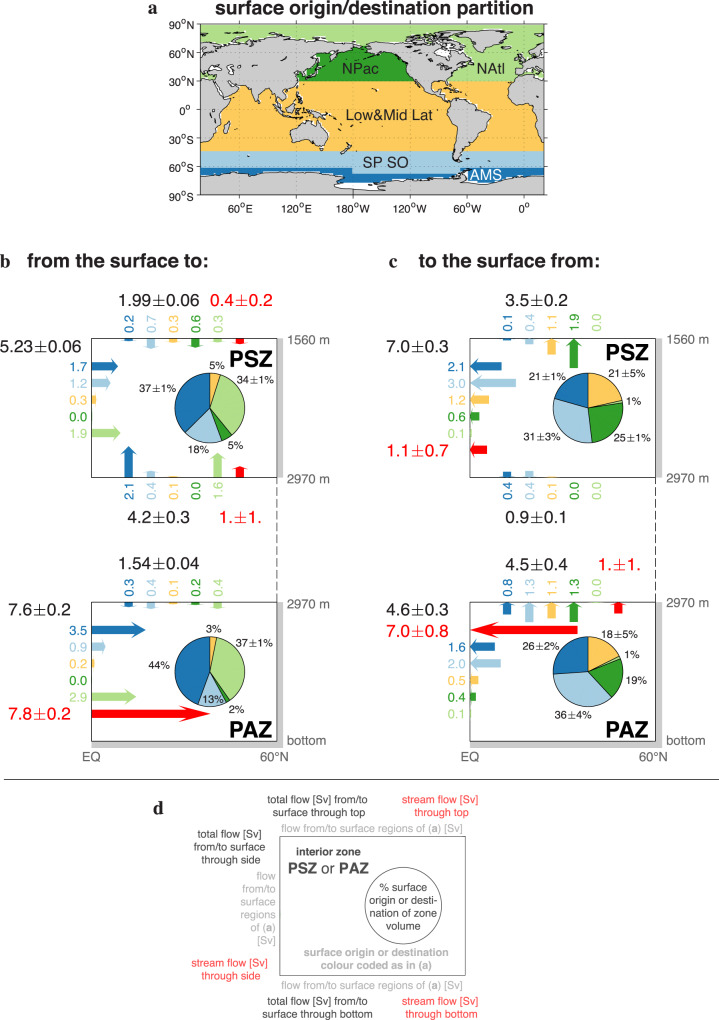
Origin and destination partitioned flow rates and interior zone volumes. Surface origin and destination partition (**a**) of the surface-to-interior-zone (**b**) and interior-zone-to-surface (**c**) volume transport rates (arrows, with length to scale) in units of Sv. Parts (**b**) and (**c**) show an idealized exploded view of the Pacific Shadow Zone (PSZ) and the abutting Pacific Abyssal Zone (PAZ) beneath in latitude-depth space. The pie charts show the partition of the zone volume according to surface origin (**b**) and surface destination (**c**). The layout of the numerical values of the volume transport rates in units of Sv is given in (**d**). Arrows and pie charts are color coded as in (**a**) according to surface region: Antarctic marginal seas (AMS, dark blue), subpolar Southern Ocean (SP SO, light blue), low and mid latitudes (Low&Mid Lat, amber), the northern Pacific (NPac, dark green), and the northern Atlantic (NAtl, light green). The surface-to-interior transport rates are plotted at the face of the interior zones (PSZ or PAZ) with which surface-origin-labelled water makes first contact, and the interior-to-surface transport rates are plotted at the face where surface-destination-labelled water had last contact with the interior zones. Colored (non-red) numbers give the magnitude (Sv) of the correspondingly colored arrows that are ordered by region; there is no significance to their position on a given zone face. For a given zone face, the black transport rates are the sum of the (non-red) colored rates. The red arrows represent the volume flow rates through the faces of the interior zones; red numbers indicate the corresponding flow rate (Sv). To emphasize the deep coherent overturning traversing the PAZ (cf. Fig. 2), the flow rate across the vertical face of the PAZ is partitioned into the inflow below and the outflow above neutral density *γ* = 28.12 kg m^−3^. [Note that the transports from the surface to first zone contact, or to the surface since last zone contact, are physically distinct from the local stream flows across the zone faces unconditioned on transport pathway (see Methods); consequently, in the presence of eddy diffusion there is no simple correspondence between the transport rates from and to the surface (black numbers) and the local stream flows (red numbers)].

The streamflow across the faces of the PSZ is weak (cf. Fig. 2) with rates of around 1 Sv, which is of the same order of magnitude as their half-range spread (Fig. 4b, c). By contrast, the surface-to-PSZ transport rate is 11.4 ± 0.3 Sv, which occurs primarily through the vertical (5.23 ± 0.06 Sv) and bottom (4.2 ± 0.3 Sv) faces. The balancing PSZ-to-surface transport is also largest through the vertical face (7.0 ± 0.3 Sv), with additional transport through the top face (3.5 ± 0.2 Sv). The fact that there is substantial transport to and from the PSZ in the absence of strong coherent overturning underscores the importance of isopycnal eddy diffusion through its equatorial side and diapycnal diffusion through its top and bottom faces.

As seen in Fig. 2, the streamflow across the equatorial boundary of the PAZ is organized by large-scale coherent overturning. The volume flow rate through the equatorial PAZ boundary below the maximum of the overturning stream function at neutral density *γ* = 28.12 kg m^−3^ represents an inflow of 7.8 ± 0.2 Sv (Fig. 4b), while above *γ* = 28.12 kg m^−3^ there is an outflow of 7.0 ± 0.8 Sv (Fig. 4c). The horizontal transport rates through the vertical face of the PAZ, both from and to the surface, are of a similar magnitude as these deep overturning rates. This and the fact that transport to the PAZ from the surface occurs mostly through its lower half (roughly below *γ* = 28.1 kg m^−3^), while transport from the PAZ to the surface occurs mostly through the upper half (quantified below), underscore the importance of horizontal advection for the PAZ. The PAZ-to-surface transport rate of 4.5 ± 0.4 Sv through the PAZ top face is about three times as large as the corresponding surface-to-PAZ transport. The fact that PAZ-to-surface transport through the PAZ top face must pass through the largely advectively decoupled PSZ indicates that this transport is controlled by diapycnal diffusion.

**Fig. 5:**
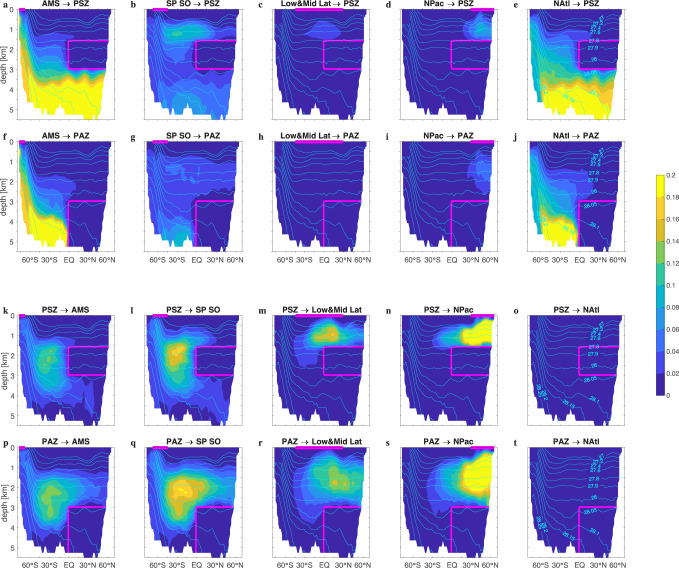
Pacific zonal mean path densities. Pacific zonal averages of the surface-to-interior path density (**a**–**j**) and of the interior-to-surface path density (**k**–**t**). (The path density is the dimensionless local fraction of water in origin-to-destination transit – see Methods.) Surface origin or destination regions as indicated in the titles and defined in Fig. 4a: Antarctic marginal seas (AMS), subpolar Southern Ocean (SP SO), low and mid latitudes (Low&Mid Lat), the northern Pacific (NPac), and the northern Atlantic (NAtl). The magenta outlines indicate the Pacific Shadow Zone (PSZ) or the Pacific Abyssal Zone (PAZ) and the part of the surface regions within the Pacific, while the cyan contours are Pacific zonal mean neutral density (−1000) in units of kg m^−3^. A generic feature of the A → B path density in the presence of diffusion is that is goes to zero close to A and close to B, where most water undergoes either A → A or B → B return trips^72^.

In Fig. 4, we have partitioned both the transport rates as well as the water volume of the interior zones according to where water was last ventilated at the surface (for surface-to-interior transport, Fig. 4b) or according to where it will next be re-exposed to the atmosphere (for interior-to-surface transport, Fig. 4c). To quantify the spatial structure of the transport pathways, we computed the path density which is the local fraction of water in transit since last contact with a specified origin region to next contact with a specified destination region^18^ (see Methods). The zonal-mean path densities are plotted in Fig. 5. Surface origin/destination is defined in terms of the 5 broad regions of Fig. 4a. The precise boundaries of the regions do not matter as the ventilation and re-exposure patterns are highly localized within these regions (Fig. 3a–d is plotted on a logarithmic scale; e.g., within the northern Pacific region ventilation occurs predominantly at mid-latitudes and in the western marginal seas while re-exposure occurs predominantly in the subarctic Pacific.) The volumetric partitioning (pie charts of Fig. 4) is qualitatively similar to the flux partitioning (colored arrows of Fig. 4) because the mean transport timescales vary by at most 50% from region to region (Fig. 3g–j). We refer to water last ventilated in the North Atlantic as North Atlantic Deep Water (NADW) and water last ventilated along the Antarctic margin as AABW. Note that this definition of water masses differs from that provided by an optimal admixture of pre-defined quasi-conservative source properties or “end members”^44,45^.

NADW and AABW are the dominant volumetric contributors to the ventilation of both the PAZ and PSZ (each contributing roughly 35–40% of the water volume of each zone), with the bulk of the remaining 20–30% coming from the subpolar Southern Ocean (mode/intermediate waters) (pie charts of Fig. 4b). NADW is about equally important for both the PAZ and PSZ, while AABW contributes relatively more to the PAZ, and mode/intermediate waters contribute relatively more to the PSZ, consistent with Fig. 3e, f. The low&mid-latitude and northern Pacific regions each contribute 5% to the PSZ volume and roughly 3% to the PAZ volume. This is consistent with some mode waters formed in the poleward flanks of the low&mid-latitude region mixing to depth (cf., Fig. 3a, c), observed deep and intermediate-water formation in the marginal seas of the western North Pacific^46,47^, and downward (in addition to upward; see below) diffusive transport in the subarctic Pacific.

The dominant surface-to-PAZ transport (Fig. 4b) through the vertical PAZ face at the equator is primarily due to AABW and NADW, both of which are transported to the PAZ mostly at densities greater than *γ* = 28.1 kg m^−3^ (Fig. 5f, j). The surface-to-PSZ transport (Fig. 4b) through the PSZ base is similarly dominated by AABW and NADW because all water entering the PSZ from below must pass through the PAZ (Fig. 5a, e). The surface-to-PSZ transports through its side and bottom faces have similar AABW and NADW contributions, but the transport through the side also contains a mode/intermediate component of comparable magnitude (Fig. 4b). Thus, the greater importance of mode/intermediate waters for ventilating the PSZ relative to the PAZ is due to quasi-horizontal isopycnal transport. Mode/intermediate waters enter the PSZ primarily through its top corner, with a small amount entrained into AABW entering from below (Fig. 5b). Most water in transit to the interior zones from the subpolar Southern Ocean can be found in the *γ* = 27.6–27.8 kg m^−3^ neutral-density range or for *γ* > 28.1 kg m^−3^ (Fig. 5b, g).

Volumetrically, the dominant destination regions for re-exposure are the Southern Ocean, the tropical oceans, and the subarctic Pacific (pie charts of Fig. 4c, maps of Fig. 3b, d). As a re-exposure destination, the northern Atlantic makes a negligible (~1%) contribution to either interior zone. For both the PSZ and PAZ, the Southern Ocean is the destination for more than half the water (52% and 62%, larger for the denser PAZ). The remainder is roughly equally split between water destined for re-exposure in low latitudes and in the subarctic Pacific.

For re-exposing PAZ water back to the surface (Fig. 4c), horizontal transport out of the side with the overturning and vertical transport out of the top are roughly equally important. The transport from the vertical PAZ face at the equator is driven by advection with the upper, lower-density branch of the overturning (28.05 ≲ *γ* ≲ 28.12 kg m^−3^). As PAZ water is transported isopycnally, it establishes a core between roughly *γ* = 27.9 and 28.0 kg m^−3^ and diffuses diapycnally into both lighter and denser layers that outcrop in the subpolar Southern Ocean and in the Antarctic margin, respectively (Fig. 5p, q). Transport rates from the vertical PAZ face to the Southern Ocean are roughly twice as large as transport rates from the top PAZ face to the Southern Ocean, with about half of the transport from the top destined for tropical and subarctic Pacific re-exposure (Fig. 4c). The required dianeutral diffusion on the way to non-Southern Ocean re-exposure (Fig. 5r, s) is manifest by several-hundred-years older mean re-exposure times (Fig. 3j).

The re-exposure of PSZ water (Fig. 4c) takes place primarily through its side face via isopycnal transport to the Southern Ocean with a core between roughly *γ* = 27.8 and 28.0 kg m^−3^ (Fig. 5k, l). Isopycnally transported water from the PSZ top and upper corner diffuses and upwells to lighter density to access the thermocline circulation (Fig. 5m, n) which delivers this water to equatorial (~2.3 Sv) and subarctic Pacific upwelling (~2.5 Sv). Mean re-exposure times Γ^*↑*^ for these waters are around 1100 years, roughly 500 years less than for PAZ-water that first needs to diffuse across the PSZ to get to the same surface destinations (Fig. 3h, j).

## Discussion

**Fig. 6:**
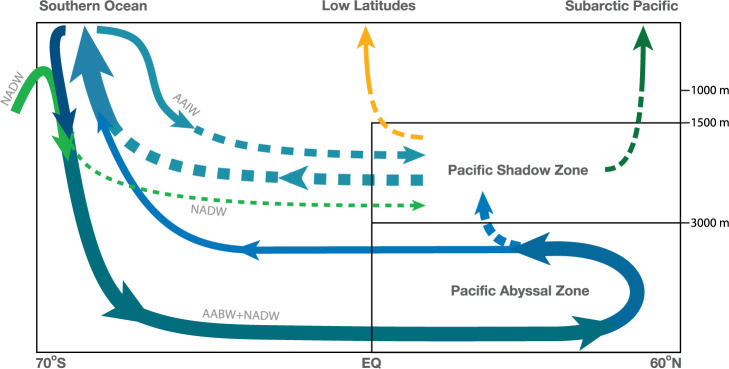
Deep Pacific ventilation schematic. Schematic summarizing the major transport pathways by which the Pacific Shadow Zone (PSZ) and the Pacific Abyssal Zone (PAZ) of the North Pacific exchange water with the surface ocean. Solid lines indicate overturning-dominated transport while dashed lines indicate diffusion-dominated transport. Bright green indicates North Atlantic ventilated water (NADW), while blue tones indicate waters last ventilated in the Southern Ocean (AABW and Antarctic intermediate water (AAIW)) or destined for re-exposure in the Southern Ocean. Amber and dark green arrows illustrate shadow-zone waters bound for re-exposure in low latitudes and in the subarctic Pacific, respectively. The thickness of the arrows scales approximately with the volume flow rate of each transport pathway, with a reference value of 7.3 Sv for the inflow of high-latitude-sourced water into the PAZ (thick solid blue-green arrow, a mixture of AABW and NADW). For the transport timescales associated with the pathways illustrated here see Fig. 3g–j. Like all circulation schematics, this picture is a simplification; for a detailed, rigorous quantification of the transports refer to Figs. 4 and 5.

Our analysis allows us to assess the degree to which the standard conveyor^7^ and the shadowed conveyor^8,48^ paradigms capture key aspects of the Pacific deep circulation and transport. The abyssal overturning of the OCIM is vertically compressed below roughly 3 km depth with an overlying shadow zone in good agreement with the shadowed conveyor paradigm^8^. The OCIM data-assimilated PMOC does not have a coherent, advectively dominated 10–15 Sv of AABW upwelling to around 1.5 km depth as inferred by geostrophic inverse models^13,14^ and suggested by typical conveyor schematics^7,11,13^. Our results therefore inspire a revamped schematic of the ventilation of the deep North Pacific which emphasizes the presence of a diffusively ventilated shadow zone above vertically compressed overturning that ventilates abyssal waters (Fig. 6).

The 11 Sv ventilating the PSZ occur mostly through quasi-horizontal isopycnal diffusion across the equator and via diapynal diffusion from below, while transport to surface re-exposure occurs mostly isopycnally across the equator and diapycnally through the top face of the PSZ (Fig. 6). The upwelling branch of the standard conveyor paradigm must therefore be interpreted broadly as representing the Lagrangian pathways of diapycnally diffusing, buoyancy gaining fluid elements rather than a branch of the PMOC. However, even broadened to a Lagrangian interpretation, the standard conveyor paradigm does not capture the existence of a shadow zone and the importance of isopycnal diffusive transport into and out of it. Precisely where water in the deep Pacific was last ventilated and where it will next be re-exposed to the atmosphere is beyond the scope of either paradigm.

The lower branch of the abyssal overturning advects about 8 Sv of predominantly AABW and NADW into the PAZ, the region below the PSZ (Fig. 6). The upper branch advects PAZ water primarily back to the Southern Ocean surface, but about 4 Sv diffuse to lighter density classes in the PSZ. This diffusive pathway, highlighted in the schematic of Fig. 6, gives PAZ waters access to the diffusively driven transport pathways that return PSZ waters to the low-latitude and subarctic Pacific surface ocean, in contrast to the widely held view that Pacific deep waters upwell exclusively in the Southern Ocean^49^. In fact, only 52% of PSZ water is re-exposed in the Southern Ocean, while 21% is re-exposed in low latitudes and 25% in the subarctic Pacific. The importance of the subarctic Pacific as a significant re-exposure destination for North Pacific deep waters is not captured by existing conveyor paradigms but is consistent with observational analyses showing that the subarctic Pacific has direct access to the abyssal nutrient supply through vertical mixing^50^ and with recent analysis of radiocarbon^51^ showing surface ^14^C depletion in this region linked to upwelling deep water.

Whereas the standard conveyor paradigm for the Pacific emphasizes the transformation of AABW to PDW, our analysis additionally highlights the importance of NADW in ventilating the deep Pacific (Fig. 6). The PSZ is fed diapycnally from below and isopycnally from the side by AABW and NADW in roughly equal measure with additional isopycnal input of mode/intermediate waters. Overall, our results underline that isopycnal diffusion is a critically important control on ventilation^52,53^.

The global surface patterns of ventilation and re-exposure are very similar for the PSZ and PAZ. The main conduits into the ocean interior are the high-latitude deep-water formation regions, while the main conduits out of the interior are the subpolar oceans and tropical upwelling, regardless of destination or origin in the deep ocean. The differences between the PSZ and PAZ manifest as subtle shifts in the relative importance of key surface regions that are isopycnally connected to these interior zones: mode/intermediate water formation is more important for PSZ ventilation, while Antarctic marginal seas are more important for the re-exposure of the denser PAZ.

Water in the PSZ has the ocean’s oldest ideal mean age (>1400 years) and is thus effectively shielded from ventilation. However, the water in the PSZ is not isolated the longest from re-exposure to the atmosphere. The longest re-exposure times (>1400 years) lie in the PAZ beneath the PSZ because much of the re-exposure of PAZ water occurs though diapycnal diffusion into the overlying shadow zone (Fig. 6) where the slow paths of the diffusive conveyor bring these waters to the surface. This underscores that access to a strong overturning circulation does not guarantee rapid transport.

By providing a more comprehensive, quantitative description of how deep North Pacific waters are ventilated and ultimately re-exposed to the surface, our analysis reconciles the two paradigms of the overturning circulation as capturing distinct aspects of the deep Pacific circulation. While the shadowed conveyor gives a better description of the vertically compressed PMOC, the standard paradigm, broadly interpreted, captures the slow diffusively dominated paths through the shadow zone. Our revamped ventilation schematic explicitly separates these two pathways while also highlighting the connectivity of the abyssal and shadow zones through diapycnal diffusion as well as the substantial diffusion-mediated transport of deep North Pacific waters to the low-latitude and subarctic Pacific surface (Fig. 6).

The detailed transport pathways and timescales revealed here have important implications for ocean biogeochemistry. About a quarter of the shadow zone eventually re-emerges in the subarctic Pacific bringing with it old nutrients to sustain biological production and pelagic ecosystems. For sequestering anthropogenic carbon, both the PSZ and the PAZ beneath it have long residence times: the PSZ has the slowest access to ventilation, while the PAZ has the slowest access to re-exposure. Poor ventilation and high carbon accumulation make the PSZ particularly sensitive to any future changes in the biological pump that drive increased oxygen demand. Importantly, the shadowed conveyor paradigm with leading-order diffusive transports allows for consistent interpretations of observed tracer distributions in the Pacific. This holds not only for age tracers such as Δ^14^C but for any tracer with a sufficiently simple source/sink structure. For example, the observed silicic-acid distribution^39^ has high concentrations in the PSZ but lower concentrations in the PAZ. This distribution is consistent with the advective flushing of deep regenerated silicic acid out of the PAZ and with the accumulation of silicic acid injected into the poorly ventilated shadow zone, but difficult to reconcile with the traditional view of deep Pacific overturning. Our analysis here applies to the current state of the ocean as the OCIM assimilates observational climatologies. Similar analyses could be applied to prognostic ocean circulation models^19,54^ in order to illuminate the roles of Pacific overturning and ventilation in climate change.

## Methods

### OCIM

The ocean circulation inverse model (OCIM) is a three-dimensional dynamical ocean model that assimilates ocean tracer data to estimate the climatological mean state of the ocean circulation^2,4,23^. The OCIM solves linearized steady-state momentum equations with neglected nonlinear terms and any discretization errors assigned to an adjustable forcing field. The equations are discretized on an Arakawa B grid^55^ with horizontal levels, and the observed density distribution is prescribed. Here, we update the most recent 24-layer version, OCIM2^4^, to an improved 48-layer version OCIM2-48L. OCIM2-48L assimilates six different circulation tracers: potential temperature (Θ), salinity (S), CFC-11, CFC-12, natural radiocarbon (Δ^14^C), and natural *δ*^3^He. Annual mean objectively analyzed observations of Θ and S are taken from the 2013 World Ocean Atlas^56,57^. CFC-11 and CFC-12 measurements were taken from version 2 of the Global Ocean Data Analysis Project (GLODAPv2^58^). For Δ^14^C, we use the same compilation of observations used for OCIM2, which includes data from GLODAPv2, a compilation of Δ^14^C measurements from surface corals^59^, historical Δ^14^C measurements^60^, and a collection of pre-bomb surface ocean Δ^14^C measurements from the 14CHRONO database (available at http://calib.org/marine/). OCIM2-48L uses the same set of *δ*^3^He observations as OCIM2, which includes *δ*^3^He observations from GLODAPv2 and from a transect in the Eastern Tropical Pacific^61^. The Δ^14^C and *δ*^3^He observations were processed using exactly the same procedure as for OCIM2, which includes screening for and removal of observations that are potentially contaminated by bomb radiocarbon and tritiogenic ^3^He. OCIM2-48L also assimilates the climatological average air-sea heat and freshwater fluxes from the NCEP reanalysis for the period 1980–2009^62^ as well as the mean dynamical sea surface topography from the AVISO data product for the period 1993–1999 (release MDT-CNES_CLS09).

These observations are assimilated into OCIM2-48L by adjusting model parameters to minimize a quadratic cost function that measures the misfit between model and observations^2,4,23^. For the CFCs, the time-dependent surface boundary conditions are propagated to the time and location of the GLODAPv2 measurements. An adjoint approach^63,64^ is used to determine the optimal model parameters that minimize the misfit cost function. As for OCIM2, these parameters include (i) a set of parameters to adjust the local geostrophic momentum balance to mimic unresolved physics and to account for model discretization errors, (ii) a set of parameters to adjust the restoring temperature and salinity used for simulating air-sea heat and freshwater fluxes at the sea surface, (iii) a set of parameters to adjust the local mantle ^3^He injection rate along mid-ocean ridges, and (iv) a single parameter to control the global relationship between gas-transfer velocity and wind speed, using a quadratic wind-speed dependence^65^. OCIM2-48L uses an additional set of adjustable parameters that controls the local value of the isopycnal diffusivity. Like previous OCIM versions, the parameters and circulation are time-invariant and thus neglect temporal (e.g., seasonal or interannual) variability. The model thereby yields a climatological mean-state estimate of the combined advective-diffusive tracer transport.

OCIM2-48L includes several updates and improvements to better represent the deep Pacific ventilation. First, the vertical resolution has been increased from 24 to 48 levels. The thickness of the layers ranges from about 10 m near the sea surface to 300 m in the abyssal ocean. This is important for better resolving the vertically compressed overturning of the deep Pacific. The horizontal resolution remains at 2 degrees, which is sufficient for resolving the large-scale circulation of the Pacific. Second, the vertical diffusivity *κ*_⊥_ in OCIM2-48L is parameterized using a recently developed global model of tidal energy dissipation^25^ due to breaking internal waves generated by tides flowing over uneven topography. This formulation of *κ*_⊥_ replaces the less realistic constant or depth-enhanced background diffusivity used in previous OCIM versions. Like previous versions of the OCIM, OCIM2-48L has enhanced vertical diffusivities in the surface mixed layer that are parameterized using the KPP scheme^66^. However, OCIM2-48L prescribes a climatological annual mean mixed-layer depth^67^ rather than the monthly maximum mixed-layer depth used in previous versions^4,23^. Finally, to account for buoyancy gain by direct geothermal heating, we have included a recent estimate of the geothermal seafloor heat-flux distribution^68^.

The high fidelity of OCIM2-48L-assimilated tracer distributions to the observations is quantified in Table 1 in terms of the root-mean-square error (RMSE) and Pearson’s correlation coefficient *r*, for both the global and Pacific-basin fields. The improved parameterization of diffusivity and higher vertical resolution result in RMS errors that are 15–50% smaller than those for OCIM2^4^. For all tracers, the fit in the Pacific is slightly better than the global fit, making OCIM2-48L well-suited for quantifying deep Pacific transport.

**Table 1:** OCIM2-48L Model–Observation RMSE and Pearson’s *r* for data-assimilated circulation tracers.

tracer	RMSE	RMSE	*r*	*r*
	global	Pacific	global	Pacific
Θ	0.34 ^∘^C	0.32 ^∘^C	0.997	0.998
S	0.038 psu	0.027 psu	0.983	0.992
CFC-11	0.22 pmol kg^−1^	0.17 pmol kg^−1^	0.977	0.979
CFC-12	0.11 pmol kg^−1^	0.08 pmol kg^−1^	0.979	0.983
Δ^14^C	3.9‰	3.6‰	0.995	0.995
*δ*^3^He	0.58%	0.46%	0.997	0.998

### Origin-to-destination water-mass fractions, path densites, flow rates, and transit times

We track water from origin to destination by labeling the water with a passive tracer. The OCIM’s steady discretized advection-diffusion flux-divergence operator is organized into a matrix **T**, and all gridded scalar fields are organized into corresponding column vectors, so that the generic tracer equation for concentration *χ* is ∂_*t*_*χ*  + **T***χ* = *S*, where *S* are the discretized source/sink terms. Because OCIM flow is steady, **T** is time-independent.

For the calculations here, we define 3 regions in terms of their discretized mask vector: the global surface layer Ω_*s*_, a subregion of the surface Ω_*a*_, and an interior region Ω_*b*_ (the PAZ or PSZ). (The entries of these masks are zero or unity depending if the corresponding grid point lies in the region or not.) We then calculate the local fraction *G*_*a*_ *dt* of water that had last contact with Ω_*a*_ during time interval (−*dt*, 0] without having had contact with Ω_*s*_ and Ω_*b*_ for *t* > 0. Disallowing repeat contact with Ω_*a*_ ensures that water is labelled according to *last* Ω_*a*_ contact, which avoids overcounting and makes it possible to partition according to transit time. Disallowing contact with Ω_*s*_ ensures that integrals over the global sea surface give the net contribution from the sea surface (see also discussion of the flow rates below).

To enforce the condition of no contact with Ω_*s*_ and Ω_*b*_, we remove Ω_*a*_-labelled water as soon as it makes contact with these regions, i.e., we impose the boundary condition that *G*_*a*_ = 0 in Ω_*s*_ and Ω_*b*_ for *t* > 0. The most convenient way to enforce these boundary conditions numerically is by relaxing *G*_*a*_ to zero with a fast timescale *τ* for grid points inside Ω_*s*_ and Ω_*b*_. (For *τ* small compared to the time for transport across a typical grid box there is no sensitivity to the precise value of *τ*; we use *τ* = 10 s.) Thus, *G*_*a*_ obeys

1 $${\partial }_{t}{G}_{a}+{\bf{T}}{G}_{a}={{\bf{L}}}_{s}({{{\Omega }}}_{a}\delta (t)-{G}_{a})+{{\bf{L}}}_{b}(0-{G}_{a}) ,$$

where *δ*(*t*) is the Dirac delta function specifying the labelling-rate distribution on Ω_*a*_, and we have defined the diagonal matrices **L**_*s*_ ≡ **diag**(Ω_*s*_/*τ*) and **L**_*b*_ ≡ **diag**(Ω_*b*_/*τ*). (Note that **L**_*s*_Ω_*a*_ = Ω_*a*_/*τ* as Ω_*a*_ is a subset of Ω_*s*_.) Similarly, the fraction that had last contact with Ω_*b*_ during (−*dt*, 0], and no contact with Ω_*s*_ and Ω_*b*_ since then, is given by

2 $${\partial }_{t}{G}_{b}+{\bf{T}}{G}_{b}={{\bf{L}}}_{b}({{{\Omega }}}_{b}\delta (t)-{G}_{b})+{{\bf{L}}}_{s}(0-{G}_{b}) .$$

(Note that **L**_*b*_Ω_*b*_ = Ω_*b*_/*τ*.)

The local steady-state fractions of water that had contact with Ω_*a*_ or with Ω_*b*_ at some point in the past (without further contact with Ω_*s*_ or Ω_*b*_) are given by ${f}_{a}^{\downarrow }=\mathop{\int}\nolimits_{0}^{\infty }{G}_{a}\ dt$ and ${f}_{b}^{\downarrow }=\mathop{\int}\nolimits_{0}^{\infty }{G}_{b}\ dt$. Time integrating (1) and (2) and defining **T**_*sb*_ ≡ **T** + **L**_*s*_ + **L**_*b*_, gives

3 $${f}_{a}^{\downarrow }={{\bf{T}}}_{sb}^{-1}{{{\Omega }}}_{a}/\tau \qquad {\rm{and}}\qquad {f}_{b}^{\downarrow }={{\bf{T}}}_{sb}^{-1}{{{\Omega }}}_{b}/\tau .$$

The corresponding fractions ${f}_{a}^{\uparrow }$ and ${f}_{b}^{\uparrow }$ that will make next contact with Ω_*a*_ or Ω_*b*_ at some time in the future are obtained in the same manner but for the time-reversed adjoint flow^69^. The time-reversed adjoint of **T**_*sb*_ is given by ${\widetilde{{\bf{T}}}}_{sb}\equiv {{\bf{W}}}^{-1}{{\bf{T}}}_{sb}^{T}{\bf{W}}$, where the *T* superscript denotes matrix transpose and **W** = **diag**(*w*), with *w* being the column vector of grid-box volumes. Thus, ${f}_{a}^{\uparrow }$ and ${f}_{b}^{\uparrow }$ are simply obtained by replacing **T**_*sb*_ with ${\widetilde{{\bf{T}}}}_{sb}$ in (3). The Ω_*a*_ → Ω_*b*_ path density *η*_*a*→*b*_ (integrated over all transit times) and the Ω_*b*_ → Ω_*a*_ path density *η*_*b*→*a*_ are given by

4 $${\eta }_{a\to b}={f}_{a}^{\downarrow }\circ {f}_{b}^{\uparrow }\qquad {\rm{and}}\qquad {\eta }_{b\to a}={f}_{b}^{\downarrow }\circ {f}_{a}^{\uparrow } ,$$

where ∘ denotes the element-wise product. The path density (4) is the local fraction of water in Ω_*a*_ → Ω_*b*_ transit; previous definitions of the path density^17,18^ considered only surface-to-surface transport for which *η*_*a*→*b*_ was additionally normalized by the ocean volume.

We calculate the Ω_*a*_ → Ω_*b*_ volume flow rate Φ_*a*→*b*_ by calculating the rate with which the fast relaxation removes Ω_*a*_-labelled water over Ω_*b*_. To get the total flow rate, we volume integrate over Ω_*b*_, and to get the flow rate into a particular face of Ω_*b*_, we integrate over the grid layer of the face only. (For short *τ* a single layer gives accurate results.) Thus, the total Ω_*a*_ → Ω_*b*_ volume flow rate can be written as

5 $${{{\Phi }}}_{a\to b}=(1/{\tau }^{2}){{{\Omega }}}_{b}^{T}{\bf{W}}\ {{\bf{T}}}_{sb}^{-1}{{{\Omega }}}_{a}=(1/{\tau }^{2}){{{\Omega }}}_{a}^{T}{\bf{W}}\ {\widetilde{{\bf{T}}}}_{sb}^{-1}{{{\Omega }}}_{b} .$$

The second expression of (5) is written in terms of the time reversed adjoint, which is particularly useful if the volume flow rate is desired at every grid point of Ω_*s*_, in which case the adjoint expression can be used to find the surface map of the flux in a single step by replacing Ω_*a*_ with **diag**(Ω_*s*_), each column of which is the Ω_*a*_ mask for a single surface grid box. Note that the Φ_*a*→*b*_ flow rates are robust transport metrics that integrate the effect of advection and diffusion over all possible paths from Ω_*a*_ to Ω_*b*_ because Φ_*a*→*b*_ is the flux into Ω_*b*_ of water labelled at Ω_*a*_. To calculate Φ_*b*→*a*_ volume flow rate, we simply interchange *a* and *b* in (5). The surface flux Φ^*↓*^ of the main text is computed at a given surface point by choosing Ω_*a*_ as the corresponding surface grid box, Ω_*b*_ as the interior destination region of interest (PSZ or PAZ), and dividing by the horizontal area of Ω_*a*_ to obtain the volume flux, i.e., the volume flow rate per unit area. Φ^*↑*^ is computed similarly, but with origin and destination interchanged.

Excluding repeat contact with the origin region eliminates the flux in the opposite direction, rendering Φ_*a*→*b*_ a one-way or gross flow rate^41–43^, with the usual net volume flow rate being the residual of the gross rates for opposite directions. If the origin region is part of the surface, excluding contact with the surface elsewhere ensures that summing over surface-origin regions tiling the global sea surface gives the total one-way flow rate from the surface. (Similarly, for the case where the destination region is part of the surface, excluding surface contact elsewhere ensures partitioning the total one-way flow rate into the surface layer without overcounting.) In this study, Ω_*a*_ and Ω_*b*_ neither overlap nor abut, ensuring that the corresponding flow rates are non-singular^43,69^.

To calculate the surface maps of Ω_*b*_-volume-averaged mean transit time, we consider *G*^*a*^, which obeys the same equation as *G*_*a*_, but without relaxation to zero on Ω_*b*_. The local fraction in the ocean interior that had last surface contact in Ω_*a*_ is then given by ${f}^{\downarrow }={{\bf{T}}}_{s}^{-1}{{{\Omega }}}_{a}/\tau$, where **T**_*s*_ ≡ **T** + **L**_*s*_, and the normalized Ω_*b*_-volume-averaged transit-time distribution is given by ${\mathcal{G}}=({{{\Omega }}}_{b}^{T}{\bf{W}}\ {G}^{a})/({{{\Omega }}}_{b}^{T}{\bf{W}}\ {f}^{\downarrow })$. The corresponding mean age is given by^69^

6 $${{{\Gamma }}}^{\downarrow }=\mathop{\int}\nolimits_{0}^{\infty }{\mathcal{G}}tdt=({{{\Omega }}}_{b}^{T}{\bf{W}}\ {{\bf{T}}}_{s}^{-2}{{{\Omega }}}_{a})/({{{\Omega }}}_{b}^{T}{\bf{W}}\ {{\bf{T}}}_{s}^{-1}{{{\Omega }}}_{a})\ \ \ .$$

The numerator and denominator of this expression were efficiently computed by taking their transpose and considering all single-grid Ω_*a*_ at once, i.e., by replacing Ω_*a*_ with **diag**(Ω_*s*_). The mean Ω_*b*_-volume-averaged re-exposure time Γ^*↑*^ is obtained in the same way but with **T**_*s*_ replaced by its adjoint ${\widetilde{{\bf{T}}}}_{s}$.

## Supplementary information

Supplementary note: Diffusivity fields

## Data Availability

The OCIM2-48L output data used in this study are available in the Figshare database under accession code 10.6084/m9.figshare.14802732^70^. The global tidal energy dissipation maps used to construct the three-dimensional *κ*_⊥_ field are available in the SEANOE database under accession code 10.17882/73082^71^.
