# Supplementary material for: Diffusion controls the ventilation of a Pacific Shadow Zone above abyssal overturning
Source: Nat Commun. 2021 Jul 16;12:4348. doi: 10.1038/s41467-021-24648-x (PMC8285511; doi:10.1038/s41467-021-24648-x)
Supplement: Supplementary file 1 — Supplementary note: Diffusivity fields [file 41467_2021_24648_MOESM1_ESM.pdf]

Supplementary Information for "Diffusion controls the ventilation of a Pacific Shadow Zone above abyssal overturning"

Mark Holzer[1,*], Tim DeVries[2,3], and Casimir de Lavergne[4]

[1]School of Mathematics and Statistics, University of New South Wales, Sydney, NSW 2052, Australia.
[2]Department of Geography, University of California, Santa Barbara, CA 93106, USA.
[3]Earth Research Institute, University of California, Santa Barbara, CA 93106, USA.
[4]LOCEAN Laboratory, Sorbonne Université-CNRS-IRD-MNHN, Paris, France.
*mholzer@unsw.edu.au

1 Supplementary note: Diffusivity fields

The diapycnal diffusivity $\kappa_\perp$ used in the ocean circulation inverse model OCIM2-48L is prescribed from an energy-conserving tidal mixing scheme[1] applied to the observed climatological stratification. This scheme has been extensively compared[1] (including plots of $\kappa_\perp$) against available micro- and fine-structure observations of turbulent energy dissipation. The $\kappa_\perp$ field used here is broadly consistent with available micro-structure observations and, above 2000 m depth, with fine-structure estimates[2,3] of mixing. Deeper than 2000 m, available fine-structure estimates[3] underestimate turbulence production by a factor of roughly 5 as compared to the micro-structure data[1].

The isopycnal diffusivity $\kappa_\parallel$ is calculated by OCIM2-48L as part of the data-assimilated fit to observations. We use the small-slope approximation for the mixing tensor which includes both Gent-McWilliams skew diffusion[4] and Redi isoneutral diffusion[5] with equal magnitudes for their diffusivities[6] denoted here as $\kappa_\parallel$. Below the mixed layer, the resulting $\kappa_\parallel$ has a global mean of 647 ± 16 m^2 s^{-1} with spatial variations ranging from 134 ± 2 to 4108 ± 28 m^2 s^{-1}. The Pacific zonal mean (Sup. Fig. 1a) of the OCIM2-48L $\kappa_\parallel$ has its highest values in the strongly eddying high-latitude baroclinic regions where $\kappa_\parallel$ exceeds 2000 m^2 s^{-1}, broadly consistent in the upper 2000 m with high-resolution modelling[7] and observation-based estimates from linear stability analysis[8], Argo float dispersion[9], and extended mixing-length theory[10]. The half-range of $\kappa_\parallel$ across the three optimized states with $1\times\kappa_\perp$, $2\times\kappa_\perp$ and $\frac{1}{2}\times\kappa_\perp$ is less than roughly 15% of the unperturbed ($1\times\kappa_\perp$) values (Sup. Fig. 1b).

A note of caution for interpreting the OCIM2-48L-derived $\kappa_\parallel$ field is in order. The OCIM2-48L $\kappa_\parallel$ is enhanced by a few hundred m^2 s^{-1} over the global mean throughout the tropical water column (Sup. Fig. 1a,b) and near the bottom throughout the basin (Sup. Fig. 1c), but does not display the strong maximum in the equatorial ocean above $\sim$500 m depth seen in the observational estimates. We attribute this difference to the approximate dynamics of OCIM2-48L. Like other versions of the OCIM[11–13], OCIM2-48L solves the linearized momentum equations with the neglected nonlinear terms and any discretization errors assigned to an adjustable forcing field of "error" terms. The model jointly adjusts the error-forcing field and the isopycnal diffusivity to provide advective-diffusive transport that matches the linearized dynamics and tracer constraints as closely as possible. This results in high-fidelity tracer transport, but on the grid scale it is not possible to unambiguously distinguish the effects of diffusivity and advection. For example, high isopycnal diffusivity could get swapped for shear-enhanced dispersion in locally strong velocity gradients for equivalent transport. Any such effects will be most pronounced near the equator where the neglected nonlinearities, and hence the effects of the error-forcing field, are largest.

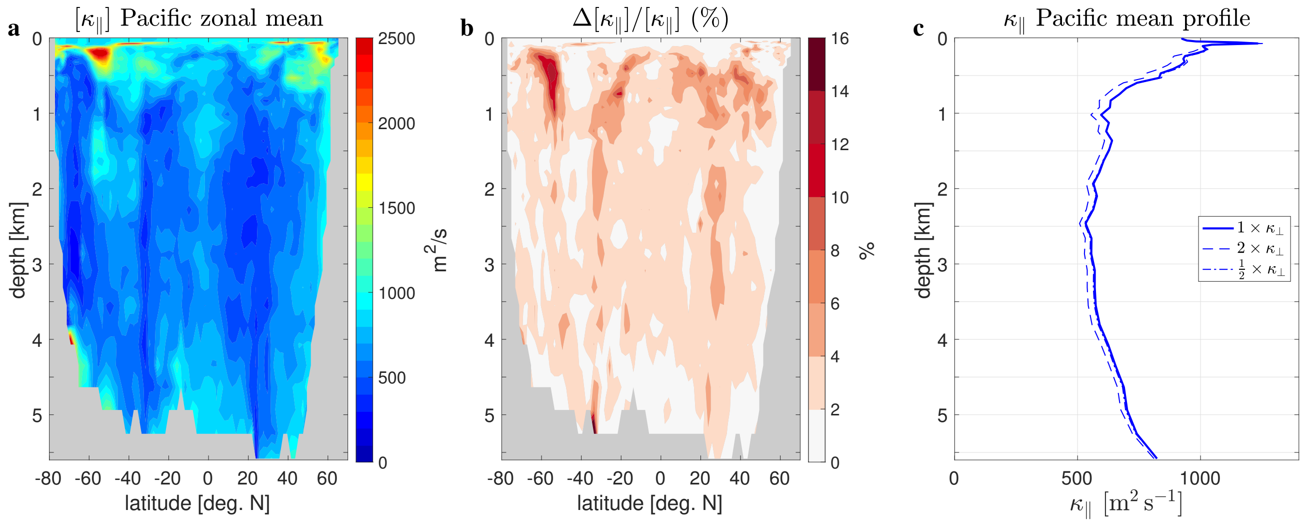

Supplementary Fig. 1: Isopycnal diffusivity in the Pacific.
The OCIM2-48L-derived isopycnal diffusivity $\kappa_{\|}$ zonally averaged across the Pacific (**a**). The half range of the Pacific zonal-mean $\Delta[\kappa_{\|}]$ across our three ocean states as a percentage of the base-state Pacific zonal-mean $[\kappa_{\|}]$ (**b**). Vertical profiles of $\kappa_{\|}$ area averaged across the Pacific (**c**) for our three ocean states: the base state and the two cases with $\kappa_{\perp}$ either scaled uniformly up or down by a factor of 2 as indicated in the legend.

References

1. de Lavergne, C. *et al.* A parameterization of local and remote tidal mixing. *Journal of Advances in Modeling Earth Systems* **12** (2020).
2. Whalen, C. B., MacKinnon, J. A., Talley, L. D. & Waterhouse, A. F. Estimating the Mean Diapycnal Mixing Using a Finescale Strain Parameterization. *J. Phys. Oceanogr.* **45**, 1174–1188 (2015).
3. Kunze, E. Internal-Wave-Driven Mixing: Global Geography and Budgets. *J. Phys. Oceanogr.* **47**, 1325–1345 (2017).
4. Gent, P. R. & McWilliams, J. C. Isopycnal mixing in ocean circulation models. *J. Phys. Oceanogr.* **20**, 150–155 (1990).
5. Redi, M. H. Oceanic isopycnal mixing by coordinate rotation. *J. Phys. Oceanogr.* **12**, 1154–1158 (1982).
6. Griffies, S. M. The Gent-McWilliams skew flux. *J. Phys. Oceanogr.* **28**, 831–841 (1998).
7. Griesel, A. *et al.* Isopycnal diffusivities in the Antarctic Circumpolar Current inferred from Lagrangian floats in an eddying model. *J. Geophys. Res.* **115** (2010).
8. Vollmer, L. & Eden, C. A global map of meso-scale eddy diffusivities based on linear stability analysis. *Ocean Modelling* **72**, 198–209 (2013).
9. Cole, S. T., Wortham, C., Kunze, E. & Owens, W. B. Eddy stirring and horizontal diffusivity from Argo float observations: Geographic and depth variability. *Geophys. Res. Lett.* **42**, 3989–3997 (2015).
10. Groeskamp, S., LaCasce, J. H., McDougall, T. J. & Rogé, M. Full-depth global estimates of ocean mesoscale eddy mixing from observations and theory. *Geophys. Res. Lett.* **47** (2020).
11. DeVries, T. & Primeau, F. Dynamically and observationally constrained estimates of water-mass distributions and ages in the global ocean. *J. Phys. Oceanogr.* **41**, 2381–2401 (2011).
12. DeVries, T. The oceanic anthropogenic CO_2 sink: Storage, air-sea fluxes, and transports over the industrial era. *Global Biogeochem. Cycles* **28**, 631–647 (2014).
13. DeVries, T. & Holzer, M. Radiocarbon and Helium Isotope Constraints on Deep Ocean Ventilation and Mantle-^{3}He Sources. *J. Geophys. Res.* **124**, 3036–3057 (2019).
